# Ni-Mg-Al Hydrotalcite-Derived Catalysts for Ammonia Decomposition—From Precursor to Effective Catalyst

**DOI:** 10.3390/molecules30051052

**Published:** 2025-02-25

**Authors:** Andrzej Kowalczyk, Martyna Zaryczny, Zofia Piwowarska, Lucjan Chmielarz

**Affiliations:** Faculty of Chemistry, Jagiellonian University, Gronostajowa 2, 30-387 Kraków, Poland

**Keywords:** ammonia decomposition, catalysis, hydrotalcite-like materials, nickel

## Abstract

A series of Ni-Mg-Al hydrotalcite-derived mixed metal oxides with different Ni/Mg ratios were prepared by the coprecipitation method followed by calcination at 600 °C. The hydrotalcite-like materials, as well as their calcined forms, were characterized with respect to structure (XRD, UV-Vis DRS), chemical composition (ICP-OES), textural parameters (low-temperature N_2_ sorption), dispersion of nickel species (H_2_-chemisorption) and nickel species reducibility (H_2_-TPR). Moreover, the process of hydrotalcite-like materials’ thermal transformation to mixed metal oxide systems in air and argon flow was studied by the TG-DTA method. The activity of the studied catalysts in the reaction of ammonia decomposition increased with an increase in nickel content in the samples. It was shown that nickel species incorporated into the Mg-Al oxide matrix segregated under conditions of reduction in a flow of H_2_/Ar mixture with the formation of metallic nickel crystallites of the average size of about 10 nm. The size of nickel crystallites was practically no change in the subsequent reduction cycles and resulted in increased catalytic activity in comparison to larger crystallites of metallic nickel (20.2–23.6 nm) deposited on Al_2_O_3_ and MgO.

## 1. Introduction

Ammonia holds great potential as both a fuel and a hydrogen carrier, offering a promising pathway towards a cleaner energy future. Ammonia can be directly used as a fuel, which is burned in internal combustion engines or used in fuel cells to generate power [1]. The advantages of ammonia as a fuel are related to no CO_2_ emission and, therefore, reducing greenhouse gas emissions compared to fossil fuels, its high power-to-fuel-to-power (PFP) efficiency [2], a large-scale production and distribution infrastructure [3], a high-octane rating of 110–130 [1] and a narrow flammability range. On the other hand, the main disadvantages of ammonia as fuel are its toxicity, emission of various pollutants upon combustion conditions, such as unburned ammonia residues and nitrogen oxides, as well as relatively low reactivity as a fuel [4]. A very promising is the application of ammonia as a hydrogen carrier. The storage and transportation of ammonia are much easier and safer than hydrogen. Ammonia can be catalytically decomposed to hydrogen and nitrogen directly before using hydrogen in fuel cells or other applications. This process can be performed on-site, eliminating the need for long-distance hydrogen transport. The advantages of ammonia in such applications are a high hydrogen density, a higher energy density than compressed hydrogen [5] and ammonia liquefication that is possible at a much higher temperature (−33 °C) than hydrogen (−253 °C) [6]. Moreover, what is important is that a well-established global ammonia industry already exists, with production plants, pipelines and transportation networks [7]. The production of green ammonia, where the process of making ammonia is 100% renewable and carbon-free, is one of the most important scientific and technical directions to develop a fully sustainable cycle of energy production [7]. Thermal dissociation of ammonia to nitrogen and hydrogen needs high operation temperatures and therefore is expensive. The temperature of ammonia cracking can be decreased, and therefore, the cost of this process can be reduced, by the application of effective catalysts [8]. The decomposition of ammonia is a two-step process involving the breaking of the N-H bond in the first step and then recombination and desorption of nitrogen atoms and hydrogen atoms into the N_2_ and H_2_ molecules, respectively [9,10]. The most active catalysts of ammonia decomposition are based on noble metals, such as ruthenium, and therefore very expensive, which makes their commercialization difficult [11]. The Ru-based catalysts are able to decompose ammonia at temperatures below 450 °C [12], but alternative catalysts with similar efficiency but cheaper have been still wanted. Much cheaper, but promising alternatives are Co-, Fe- and Ni-based catalysts [13,14], used in the form of monometallic or multimetallic systems. Their catalytic performance depends on various physical and chemical properties, such as metal dispersion [11], interaction with support [8,15] or coexistence of other components [14]. It is postulated that the rate-determining step in the process of ammonia decomposition is the recombination and desorption of N_2_ molecules [16], and, in general, the binding energy between metal and nitrogen (M-N) determines the catalytic activity of ammonia decomposition reaction [15]. Too weak M-N interactions result in limited catalytic activity in ammonia decomposition, while in the case of too strong M-N interactions, the recombination and desorption of N_2_ molecules are difficult, and therefore, the overall reaction rate is decreased. Hansgen et al. [17] calculated that the M-N binding energy necessary for the recombination and desorption of N_2_ molecules should be approximately 561 kJ/mol, while for nickel, this binding energy is about 523 kJ/mol. Thus, the M-N binding energy for metallic nickel is relatively close to the calculated optimal M-N binding energy. On the other hand, Tabassum et al. [18] postulated that recombination and desorption of N_2_ molecules are more energetically favorable on the metal–oxide (support) interfaces. Thus, the selection of suitable support and controlling the size of metal crystallites are very important issues in the design of an effective catalyst for the ammonia decomposition process.

Hydrotalcite-like materials, called also layered double hydroxides (LDHs), containing apart from magnesium and aluminum, transition metal cations, such as nickel, cobalt or iron, are reported as very promising precursors of the ammonia decomposition catalysts [19,20,21,22]. Calcination of such hydrotalcite-like materials results in materials containing highly dispersed transition metal species in a Mg-Al oxide matrix [23,24,25]. Moreover, such calcined materials are characterized by a relatively high specific surface area and porosity [26,27,28], while surface acidic/basic properties of such materials can be adjusted by changing the Mg/Al ratio in the catalyst precursors [29,30]. Therefore, hydrotalcite-like materials are very promising catalyst precursors for various reactions [31,32], including ammonia decomposition [33].

Our previous studies [14] showed much better catalytic activity of the Ni-Mg-Al, Co-Mg-Al and Co-Ni-Mg-Al hydrotalcite-derived catalysts than Ni, Co or Co-Ni supported on Al_2_O_3_ and MgO, indicating that surface acidity or basicity of supports used is less important that stabilization of deposited metal particles. The presented studies are focused on the analysis of Ni-Mg-Al hydrotalcite-like materials as precursors of the catalysts for ammonia decomposition. The main study direction has been related to the determination of the real form of such catalytic systems, obtained under conditions of calcination and pre-reduction as well as under reaction conditions. Moreover, the correlation between forms of nickel species and their activity studied catalysts was analyzed.

## 2. Results and Discussion

**Figure 1:**
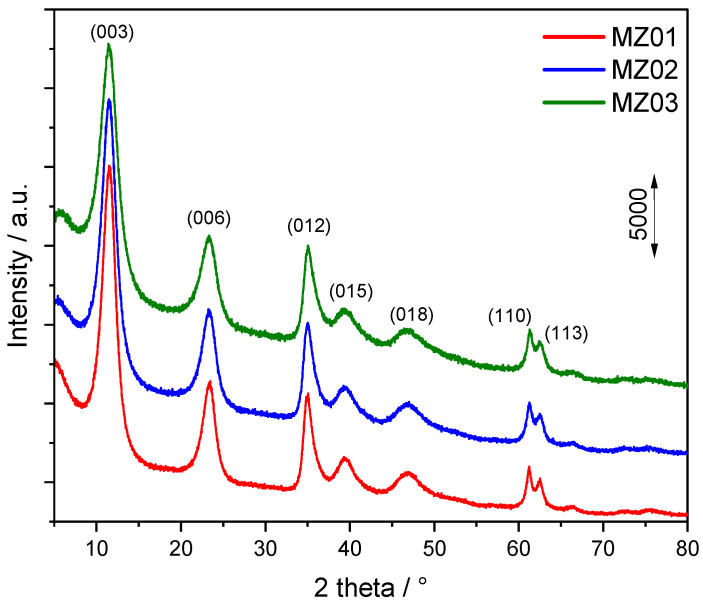
Diffractograms recorded for the Ni-Mg-Al hydrotalcite-like materials.

The sample codes as well as the intended cationic and anionic composition of the hydrotalcite-like samples are presented in Table 1, while X-ray diffraction patterns recorded for the non-calcined hydrotalcite-like samples are shown in Figure 1. In the case of all the studied samples, only reflections characteristic of the hydrotalcite structure were identified, indicating successful synthesis of the hydrotalcite-like materials without any additional crystal phases. The average size of the hydrotalcite crystals is about 5.1 nm in the case of MZ01, 4.5 nm for MZ02 and 3.9 nm for MZ03. The average size of crystallites was calculated for 003 and 006 diffraction peaks characteristic of hydrotalcite-like materials (Figure 1).

**Figure 2:**
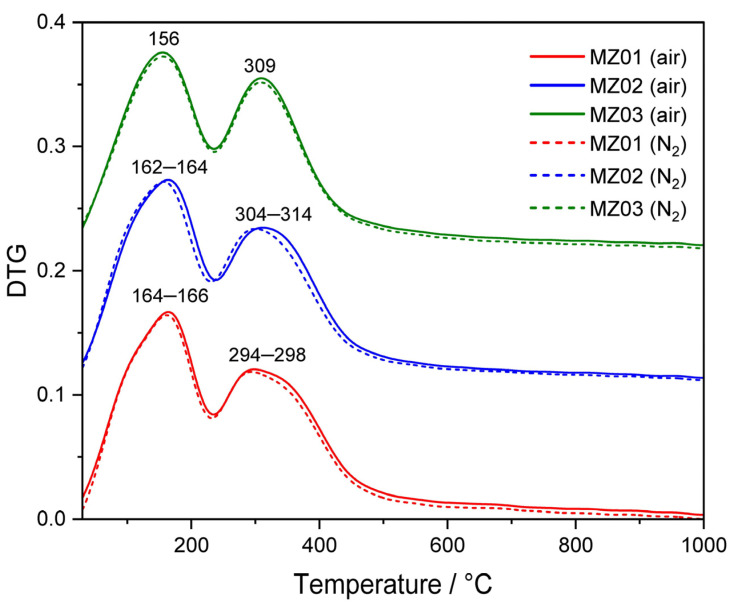
DTG profiles obtained for the thermal decomposition of hydrotalcite-like materials in flow or air (solid line) and nitrogen (dashed line).

The thermal decomposition of hydrotalcite-like materials to mixed metal oxide systems, used after reduction as catalysts, was studied by thermogravimetric analyses (Figure 2). Thermally induced decomposition of the samples was analyzed in the flow of air (solid line) as well as nitrogen (dashed line). Thermal decomposition of hydrotalcite-like materials is characterized by two main transitions: (1) the loss of interlayer water without collapse of the layered hydrotalcite structure and (2) subsequent loss of hydroxyl groups accompanied by water release from the brucite-like layers and decomposition of volatile interlayer anions, such as carbonates at higher temperatures [23,34]. The temperature ranges of these two transitions depend on the cationic composition of the hydroxide layers and interlayer anions. The first transition related to the loss of interlayer water is represented by DTG peaks at temperatures below 250 °C. The second stage of hydrotalcite-like samples’ decomposition, including dehydroxylation of the brucite-like layers as well as decomposition of interlayer anions with the evolution of their gaseous products (e.g., in the case of carbonates decomposition CO_2_ is formed), is represented by DTG peaks centered about 300 °C [23]. The DTG profiles obtained for the experiments performed in the flow of air (solid line) as well as nitrogen (dashed line) are very similar (Figure 2), indicating that the transformation of hydrotalcite-like materials into mixed metal oxides is induced by temperature, and the gas atmospheres are less important in this case. Based on the results of thermogravimetric analyses, calcination of hydrotalcite-like materials was carried out at 600 °C in an air atmosphere for 12 h.

The chemical composition of the samples calcined at 600 °C and the specific BET surface area measured for the samples calcined at 600 and 800 °C as well as after a single catalytic test are shown in Table 2. As can be seen, the real content of magnesium, aluminum and nickel is very similar to the intended values presented in Table 1. The specific BET surface area, determined for the samples calcined at 600 °C, is in the range of 311 to 334 m^2^/g. An increase in calcination temperature to 800 °C decreased the BET surface area of the samples to 200–205 m^2^/g. The samples after the catalytic test presented very similar values of specific surface area, and only in the case of the sample with the highest nickel content, MZ03, the more significant drop in BET surface area was observed (Table 2). It should be noted that the samples treated at temperatures as high as 800 °C still present a relatively high specific BET surface area.

**Figure 3:**
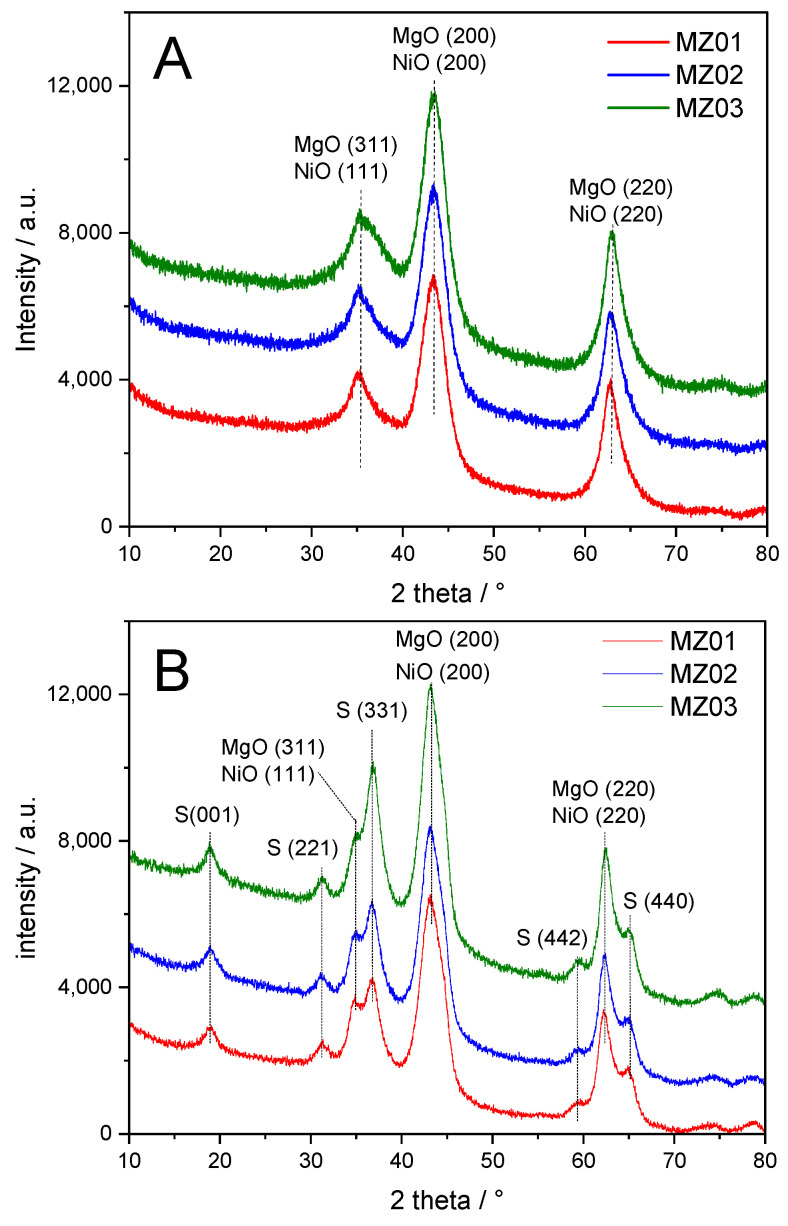

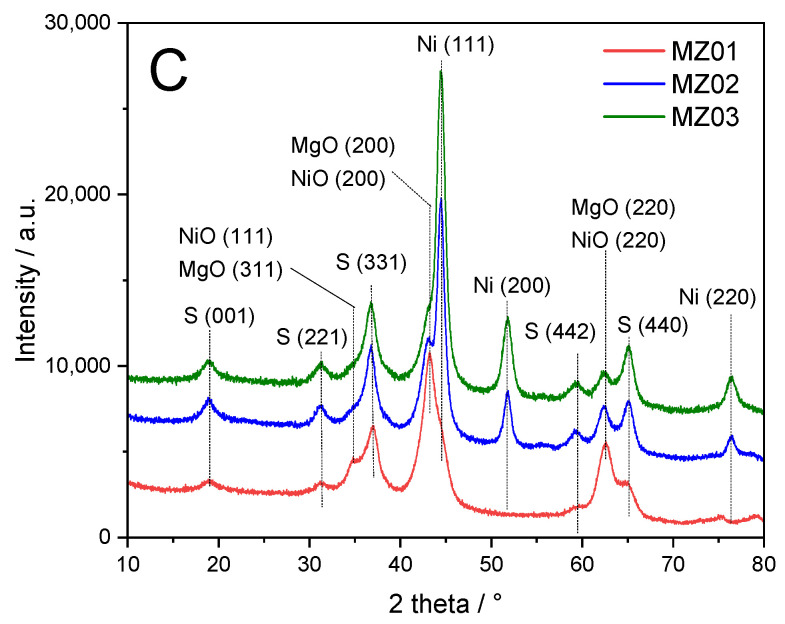
Diffractograms of the samples calcined at 600 °C (**A**) and 800 °C (**B**) as well as after catalytic test (**C**).

Diffractograms recorded for the samples calcined at 600 and 800 °C as well as after the catalytic test are presented in Figure 3. In diffractograms recorded for the samples calcined at 600 °C in an air atmosphere (Figure 3A), diffraction peaks are characteristic of periclase (MgO) and possibly also nickel oxide (reflections characteristic of NiO overlap with the reflections of MgO) [35,36]. Of course, the substitution of Ni^2+^ cations into periclase into positions of Mg^2+^ cations cannot be excluded due to similar ionic radius (Mg^2+^—72 pm and Ni^2+^—75 pm). No reflections indicating the presence of Al_2_O_3_, as a separate phase, were found in diffractograms of the calcined samples. A similar effect was reported in our previous papers and was assigned to the incorporation of Al^3+^ cations into the MgO matrix [23]. Substitution of Al^3+^ cations into the positions of Mg^2+^ cations should result in a positive charging of the matrix, which possibly is compensated by stable CO_3_^2−^ anions. Decomposition of such anions occurs at higher temperatures and is possibly represented by shoulders above 500 °C (Figure 2). An increase in calcination temperature to 800 °C (air atmosphere) resulted in the formation of spinel phases, possibly MgAl_2_O_4_, NiAl_2_O_4_ and Mg_1−x_Ni_x_Al_2_O_4_ [37,38], proved by the appearance of the specific diffraction peaks (Figure 3B). It should be noted that spinel phases coexist with periclase and possibly also NiO. Very significant changes in diffractograms (Figure 3C) are observed for the catalysts after catalytic tests (the catalysts were moved just after catalytic tests into the XRD chamber and diffractograms were recorded). It should be remembered that prior to the catalytic tests, the catalysts were reduced in a flow of H_2_/Ar mixture at 800 °C for 12 h, and catalytic tests were conducted in a flow of NH_3_/He mixture up to 800 °C. Thus, the structural changes in the samples could be induced both by high temperature as well as the presence of hydrogen or ammonia. The comparison of diffractograms presented in Figure 3A,B shows that increased temperature (800 °C) resulted in the formation of spinel phases [37,38], which are also present in the samples after catalytic tests (Figure 3C). The most intensive diffraction peaks are related to metallic nickel crystallites [39,40], which possibly were segregated from other phases. In the case of the MZ02 and MZ03 samples, the estimated average size of nickel crystallites (determined for the Ni (200) diffraction peak) was about 8.5–10.1 nm. For the sample with the lowest nickel content, MZ01, the Ni (200) reflection is not present in the diffractogram, and also other diffraction peaks characteristic of metallic nickel crystallites are less intensive compared to those identified in diffractograms recorded for the MZ02 and MZ03 samples (Figure 3C).

**Figure 4:**
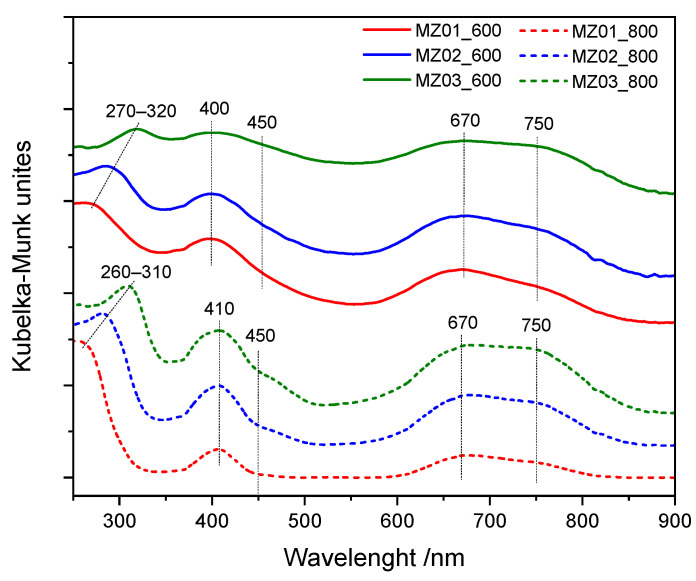
UV-Vis DR spectra recorded for the samples calcined at 600 and 800 °C.

To obtain more information about the structure of the samples, UV-Vis DR spectra were recorded for the samples calcined at 600 and 800 °C (Figure 4). The absorption band located in the range of 260–320 nm, both for the samples calcined at 600 and 800 °C, is assigned to the charge transfer O^2−^(2p) → Ni^2+^(3d) in Ni_x_Mg_1−x_O oxide [41]. The location of the band depends on the Ni concentration in such oxide. The bands centered at about 400–410, 670 and 750 nm, in spectra of the samples calcined at 600 and 800 °C, are related to the presence of Ni^2+^ cations in octahedral coordination and indicate the characteristic ^3^A_2g_ → ^3^T_1g_(P), ^3^A_2g_ → ^1^E_g_ and ^3^A_2g_ → ^3^T_1g_(F) transitions, respectively [42,43]. These bands could be assigned to Ni^2+^ species in NiO, as well as in Ni_x_Mg_1−x_O, in a wide range of concentrations, including the trace amounts of Ni^2+^ in MgO.

Comparison of the results obtained by XRD (Figure 3) and UV-Vis DRS (Figure 4) methods clearly shows that nickel in the calcined samples is present mainly in the form of NiO and Ni_x_Mg_1−x_O oxides. The diffraction peaks related to the spinel phase, observed in diffractograms of the samples calcined at 800 °C (Figure 3B), are most probably assigned to the presence of MgAl_2_O_4_.

**Figure 5:**
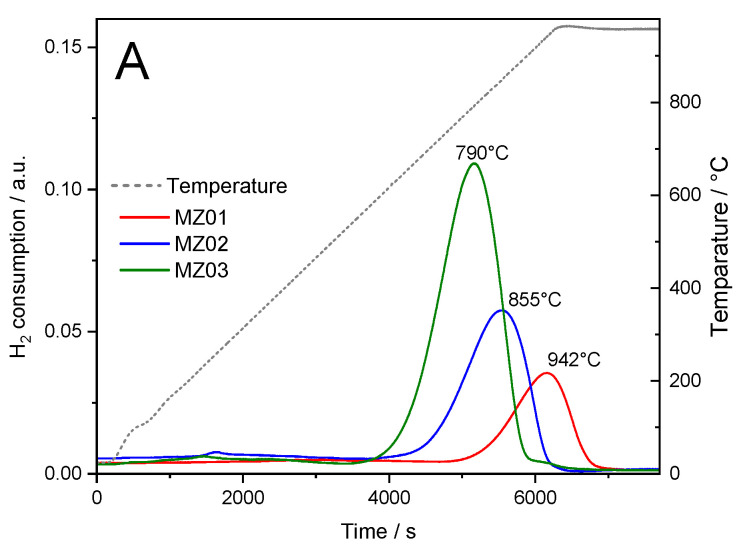

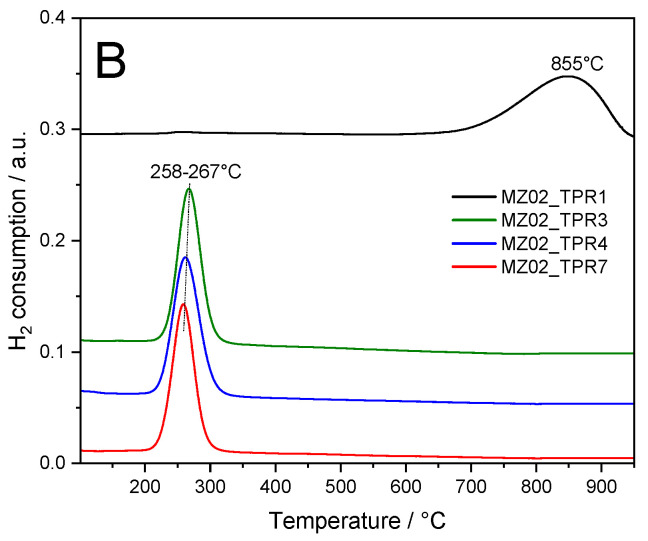
H_2_-TPR profiles of the samples calcined at 600 °C (**A**) and reduction profiles of the MZ02 sample recorded in the subsequent H2-TPR runs (**B**).

The reducibility of nickel in calcined at 600 °C hydrotalcite-like materials was studied by temperature-programmed reduction method using hydrogen as a reducing agent (H_2_-TPR). Reduction profiles, presented in Figure 5A, indicate that nickel cations present in the periclase matrix are effectively stabilized, and their reduction is observed above 600 °C [44]. An increase in nickel content results in a decrease in reduction temperature. For the sample MZ01, the maximum is located at 790 °C, for MZ02 at 855 °C and for MZ03 at about 940 °C. Su et al. [45], who studied calcined Ni-Mg-Al hydrotalcite-like materials, reported a similar effect of reduction temperature shift for the materials with the increasing Mg/Ni ratio and explained that this effect by the increase in the number of electrons on the surface of the catalyst through the doping of Mg improves the electron transfer from the carrier surface to the active metal center, thus improving the nickel species stability. Small and broad maxima at about 250–300 °C are possibly associated with the reduction of NiO aggregates weakly interacting with the Mg-Al oxide matrix [46,47]. The very low intensity of these peaks may indicate that the majority of nickel is incorporated into periclase, and only very small amounts of nickel exist in the form of NiO.

To verify the stability of nickel species in the studied catalysts, the reduction–oxidation cycles of the MZ02 sample were repeated seven times. It should be remembered that catalysts are reduced prior to the catalytic tests; thus, this final step of the catalyst activation is very important for the overall performance of the catalytic systems. The reduction profiles of the MZ02 catalyst—in the first H_2_-TPR cycle (the sample calcined at 600 °C) and in the third, fourth and seventh H_2_-TPR cycles—are shown in Figure 5B. As was already mentioned, the reduction of hydrotalcite derived sample calcined at 600 °C took place at higher temperatures and is represented by intensive maximum centered at about 855 °C. In this case, Ni^2+^ cations introduced into the periclase are reduced. Relatively high reduction temperatures indicate strong stabilization of nickel species in such a matrix [46,47]. The H_2_-TPR run was conducted in a flow of gas mixture containing 5 mol% of hydrogen diluted in argon up to 800 °C. Such high-temperature treatment of the catalyst in the flow of H_2_/Ar mixture resulted in a very significant change in the H_2_-TPR profile in the subsequent reduction cycles (Figure 5B). The high-temperature peak disappeared and a new, low-temperature maximum centered at about 255–270 °C, assigned to the reduction of NiO particles weakly interacting with support [46,47], appeared. Such significant changes in the reduction profiles indicate that Ni^2+^ cations or their reduced forms are removed from the periclase matrix and form Ni species located on the surface of the catalyst at higher temperatures of the H_2_-TPR cycle. Detailed analysis of H_2_-TPR profiles shows that the position of the reduction maximum was slightly shifted from 267 °C for the third cycle to 258 °C for the seventh cycle, which could be attributed to the weakening of the nickel species interactions with support as a result of the subsequent reduction cycles.

**Figure 6:**
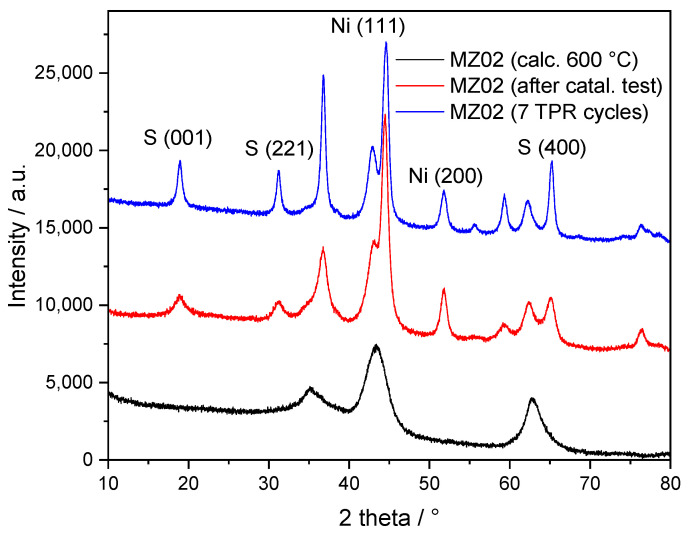
Diffractograms of the freshly calcined MZ02 sample at 600 °C as well as after catalytic tests and seven H_2_-TPR cycles.

Diffractograms recorded for the MZ02 sample, calcined at 600 °C, after the single catalytic test (reduction at 800 °C and catalytic test) and after seven H_2_-TPR cycles (to 800 °C) are shown in Figure 6. It should be noted that diffractograms of the MZ02 sample after the H_2_-TPR cycle and after the catalytic test are very similar and indicate the presence of metallic nickel and spinel phases. The average size of nickel crystallites, determined from Ni(200) reflection, is about 10.3 and 10.5 nm for the MZ02 sample after a single catalytic test and after seven H_2_-TPR cycles, respectively. Thus, an increase in nickel crystallites took place during the first catalytic cycle and in the subsequent cycles is limited.

The dispersion of nickel in the samples was analyzed by H_2_-chemisorption. The results of these studies, presented in Table 3, show that the estimated surface area exposed by metallic nickel increases with an increase in this metal content. The comparison of metallic nickel surface area to nickel content (Table 3) shows nearly the same values for all the samples, indicating that the available for the reaction nickel surface increases proportionally to its content. Based on the presented results, it can be concluded that the real catalysts operating in catalytic tests are composed of periclase, spinels (possibly MgAl_2_O_4_) and metallic nickel crystallizers.

**Figure 7:**
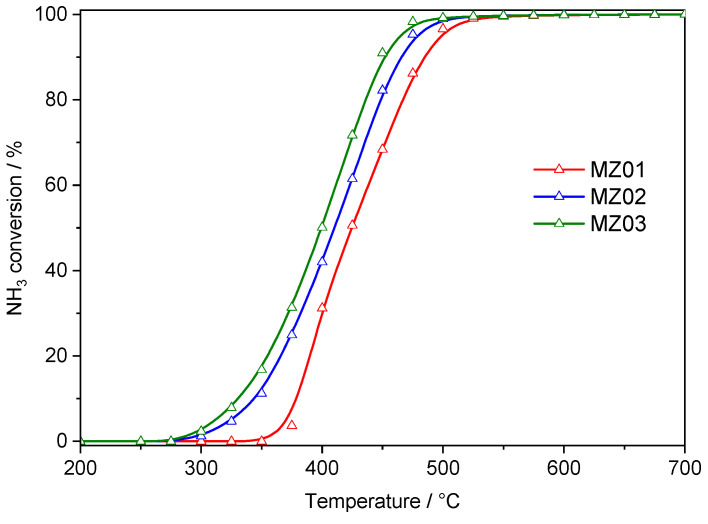
Results of the catalytic tests of the ammonia decomposition reaction.

Calcined hydrotalcite-like materials were found to be effective catalysts of ammonia dissociation with hydrogen and nitrogen (Figure 7). Ammonia decomposition started at about 300–350 °C, depending on nickel content in the catalysts. The level of 50% ammonia conversion was achieved at 424, 410 and 400 °C for MZ01, MZ02 and MZ03, respectively, while the level of 90% ammonia conversion at 485 °C for MZ01, 466 °C for MZ02 and 451 °C for MZ03. Thus, an increase in nickel content results in catalyst activation expressed by a decrease in temperatures of ammonia decomposition. The obtained results are very promising in comparison to other Ni-based catalysts reported in scientific literature. Kowalczyk et al. [14] reported an ammonia conversion level of 90% at temperatures of 680 and 580 °C in the presence of supported Ni/MgO and Ni/Al_2_O_3_ catalysts. The nickel content in these catalysts was about 5.7 wt.%, so similar to the content of nickel in the MZ01 catalysts (5.8 wt.%). Such significant differences in the catalytic activity of various Ni-catalysts are dependent on support used as well as nickel dispersion. It has been postulated that the rate-determining step in ammonia decomposition is nitrogen species recombination and N_2_ desorption, which possibly occurs on the border between nickel particles and support [40]. As was already mentioned in the case of the MZ02 and MZ03 catalysts, the average Ni crystallites size is 8.5–10.1 nm, while for Ni/MgO and Ni/Al_2_O_3_ was determined to be about 23.6 and 20.2 nm, respectively [14]. Therefore, in the case of hydrotalcite-originated catalysts, the border between nickel crystallites and Mg-Al oxide matrix is significantly larger compared to the supported Ni/MgO and Ni/Al_2_O_3_ catalysts, and recombination and desorption of nitrogen molecules should be more effective. The role of metal particle size in ammonia decomposition over monometallic and bimetallic Ni-Co catalysts was studied by Wu et al. [15]. It was postulated that optimal catalytic performance can be obtained for highly dispersed metal crystallites with an average size of 10 nm or even smaller. Thus, controlling the nickel crystallite size is very important for tailoring effective catalysts for ammonia cracking. As already mentioned, the average size of metallic nickel crystallites in the MZ02 catalysts increased from 10.3 nm after a single catalytic run to 10.5 nm after seven H_2_-TPR cycles (temperature up to 800 °C). Thus, the size of nickel crystallites is stable over extended high-temperature treatments. The results of the catalytic studies obtained for the MZ02 catalyst were compared with the Co-Mg-Al and Fe-Mg-Al hydrotalcite-derived catalysts containing very similar transition metal content (Supplementary Materials, Figure S1). It was shown that a nickel-containing catalyst (MZ02) operated at significantly lower temperatures compared to other catalysts, and therefore, Ni-based catalysts seem to be much more promising than other studied samples.

**Figure 8:**
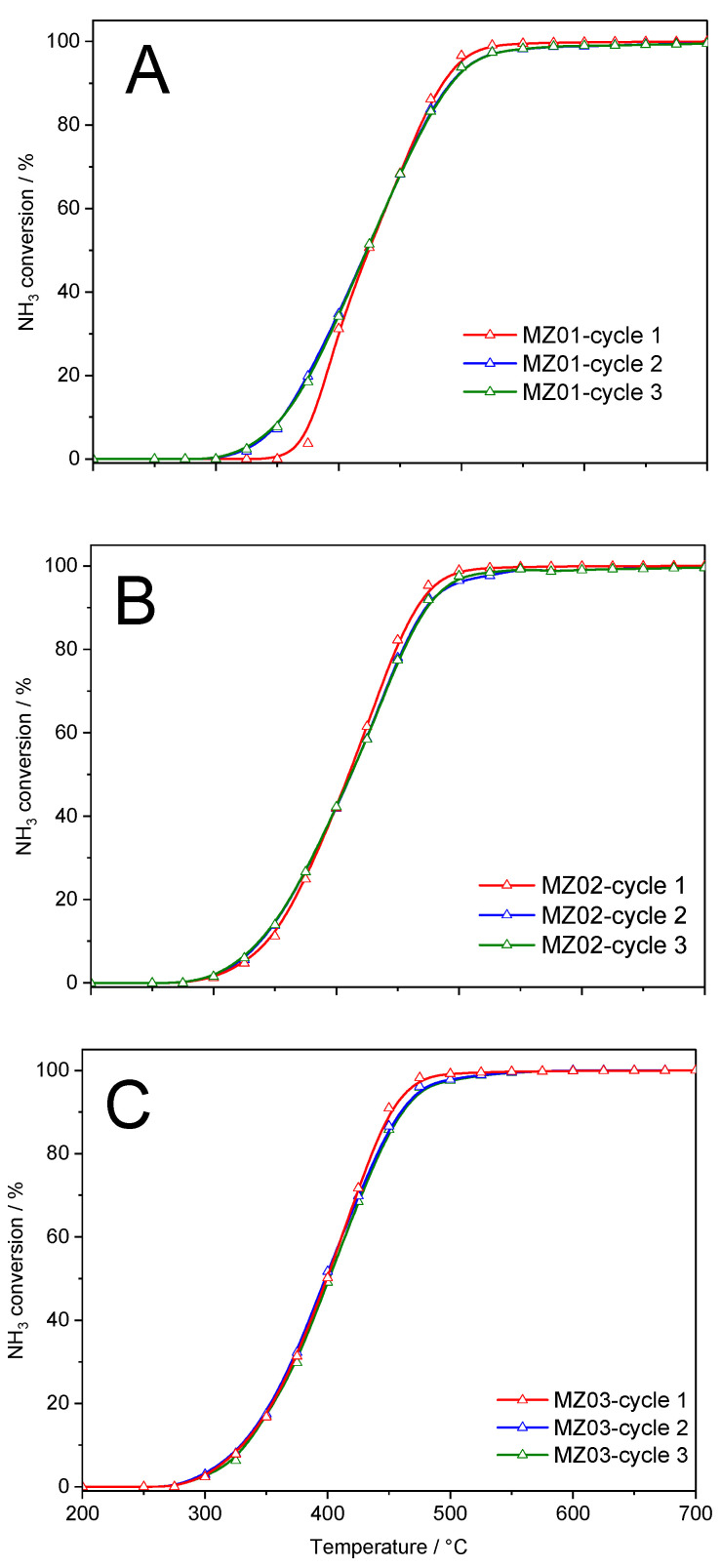
Studies of the catalyst’s stability in the subsequent catalytic cycles of ammonia decomposition reaction in the presence of MZ01 (**A**), MZ02 (**B**), and MZ03 (**C**) catalysts.

To verify the catalytic stability of the catalysts under conditions of the catalytic tests, three subsequent catalytic runs were performed for each sample (Figure 8). Prior to the first catalytic run and between subsequent runs, the catalysts were reduced at 800 °C in the flow of the H_2_/Ar mixture. In the case of the MZ01 sample, the most significant differences in the shape of the ammonia conversion profile are observed between cycle 1 and next cycles, which are very similar (Figure 8A). In cycles 2 and 3, ammonia conversion proceeded more effectively at temperatures below 450 °C and was slightly limited above 450 °C compared to the first cycle. A similar effect was observed for the MZ02 sample (Figure 8B); however, in this case, the differences in the profile shapes were less significant compared to MZ01 (Figure 8A). In the case of the most active catalyst, MZ03, the differences in the ammonia conversion profiles are observed only at higher temperatures and are expressed by slightly lower activity of the catalyst in cycles 2 and 3 compared to cycle 1 (Figure 8C). It should be noted that differences in the conversion profiles in the subsequent catalytic cycles decrease with an increase in nickel content in the samples. Possibly these differences are related to the changes in the form and aggregation of nickel species in the samples occurring under conditions of the reduction and catalytic test. As was shown by H_2_-TPR results of the calcined hydrotalcite-like samples, the reduction temperature of nickel species increased with a decreasing nickel content in the catalysts as well as increasing magnesium content (Figure 5A), indicating more effective stabilization of nickel species in such Mg-Al oxide matrix. Thus, it seems that the reduction and aggregation of metallic nickel crystallites need extended thermal treatment in the reducing atmosphere. However, it can be concluded that starting from the second catalytic cycle, for all studied catalysts, the results of the catalytic tests are fully reproducible (Figure 8). The ammonia conversion level of 90% for stabilized catalysts (cycle 3) was obtained at 492 °C for MZ01, 472 °C for MZ02 and 460 °C for MZ03, which still classify these catalysts as very promising.

**Figure 9:**
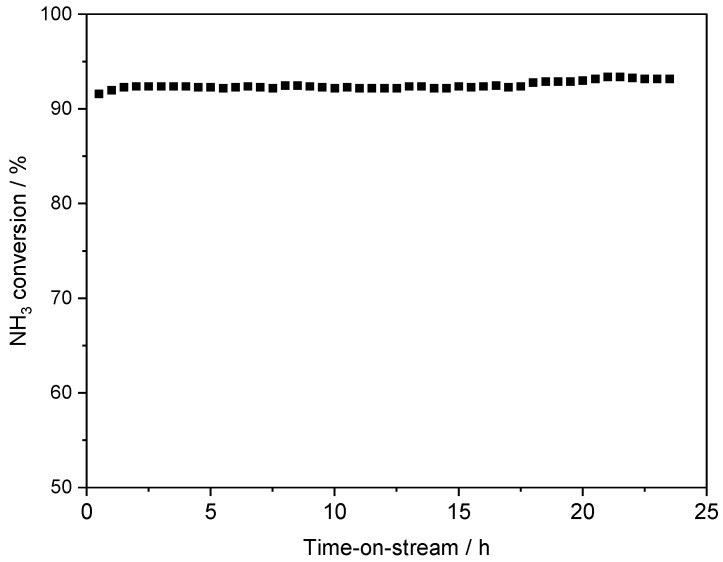
Long-term isothermal stability test for the MZ03 catalyst at 450 °C.

The stability of the MZ03 catalyst under reaction conditions was verified by an isothermal long-term stability test conducted at 450 °C for 23 h (Figure 9). As can be seen during the whole studied period, the ammonia conversion was on the same level, indicating the high stability of this catalyst under ration conditions.

## 3. Materials and Methods

### 3.1. Catalysts Synthesis

A series of Ni-Mg-Al hydrotalcite-like materials with various Ni/Mg ratios were prepared by the coprecipitation method. Aqueous solutions were prepared by dissolving Mg(NO_3_)_2_·6H_2_O (Honeywell, Seelze, Germany), Al(NO_3_)_3_·9H_2_O (ChemPur, Piekary Śląskie, Poland) and Ni(NO_3_)_2_·6H_2_O (Honeywell, Seelze, Germany) with an appropriate molar ratio to obtain materials with the intended molar composition presented in Table 1. The total metal ions concentration in a solution was 0.5 mol/L. Such solution was dropwise added to an intensively stirred solution of Na_2_CO_3_ (0.1 mol/L, POCH/Avantor, Gliwice, Poland). A constant pH of the mixture was maintained at the level of 10.0 ± 0.2 by the addition of NaOH solution (1.0 mol/L, Honeywell, Seelze, Germany). The obtained suspension was aged at 60 °C for 2 h with vigorous stirring. Then the obtained solid product was separated by filtration, washed with warm distilled water and dried overnight in air at 60 °C. In the final step, the obtained solid samples were calcined in an air atmosphere at 600 °C for 12 h. The intended and real chemical composition of the samples as well as sample codes are shown in Table 1.

### 3.2. Catalysts and Catalyst Precursors Characterization

The thermal decomposition of hydrotalcite-like material to mixed metal oxides was studied by thermogravimetric (TG) analysis using TGA/DSC 3+ (Mettler-Toledo, Columbus, OH, USA) instrument. The measurements were conducted in the temperature range of 30–1000 °C with a linear temperature increase of 20 °C/min. The TG runs were performed in a flow (80 mL/min) of synthetic air as well as pure nitrogen.

The chemical composition of the calcined hydrotalcite-like materials was determined by the inductively coupled plasma optical emission spectroscopy method (ICP-OES) using the iCAP 7400 instrument (Thermo Fisher Scientific, Waltham, MA, USA). Prior to the analysis, the solid samples were digested in a solution composed of 6 mL of HNO_3_ (67–69%, Honeywell, Charlotte, NC, USA) and 2 mL of H_2_O_2_ (30%, Avantor/POCH, Gliwice, Poland) at 190 °C using an Ethos Easy microwave digestion system (Milestone, Sorisole, Italy).

The structure of the hydrotalcite-like samples as well as their calcined forms was verified using the X-ray diffraction method. The powder diffractograms were recorded by an Aeris (Malvelm Panalytical, Westborough, MA, USA) diffractometer operating with the Cu K_α_ radiation (λ = 0.154 nm). The diffractograms were recorded in the 2θ angle range of 3–80° with a step of 0.02°. A counting time of 1 s per step and a sample rotation of 15 rpm were applied in all XRD measurements. Moreover, diffractograms were recorded for the samples just after catalytic runs.

The textural parameters of the calcined samples, such as specific BET surface area and pore volume, were determined by low-temperature (−196 °C) nitrogen adsorption and desorption measurements using a 3Flex (Micromeritics, Norcross, GA, USA) adsorption analyzer. Prior to the sorption measurements, the calcined samples were degassed under vacuum (0.2 mbar) at 350 °C for 24 h. The specific BET surface area (S_BET_) was determined using the Brunauer–Emmett–Teller (BET) model.

The reducibility of the calcined samples was analyzed by the method of temperature-programmed reduction with hydrogen (H_2_-TPR). Prior to the H_2_-TPR run, 30 mg of the catalyst was placed in the quartz flow microreactor and degassed in a flow of high purity Ar (purity class 5.0) at 550 °C for 30 min. After the microreactor was cooled down to 50 °C, the flow of pure argon was switched for a flow of gas mixture containing 5.0 mol% of H_2_ diluted in Ar (purity class of both—5.0), and the linear temperature increase (10 °C/min.) was switched on. The H_2_-TPR run was performed at 950 °C with a flow of H_2_/Ar mixture of 10 mL/min. The water vapor was removed reactor downstream by cold trap, while hydrogen consumption was analyzed by a thermal conductivity detector—TCD (TCD3, Valco, Houston, TX, USA) connected directly to the reactor outlet via a heated line.

The form and aggregation of nickel species in the samples were verified by UV-Vis DR spectroscopy. The spectra of the samples were recorded using an Evolution 220 (Thermo Scientific, Waltham, MA, USA) spectrophotometer equipped with integrating sphere ISA-220 operating in a reflectance mode. The spectra were recorded in the range of 190–900 nm, with a resolution of 1 nm.

Dispersion of nickel was determined by pulse H_2_ chemisorption method using a microreactor system. The sample of 100 mg was placed in a flow microreactor and reduced in a flow of 5 mol% H_2_ diluted in Ar (10 mL/min). In the next step, the microreactor was cooled to 50 °C and then flushed in a pure argon flow in the linear temperature program of 50–400 °C to remove residual hydrogen from the sample surface. After cooling the sample to 100 °C in a flow of argon, the pulses of hydrogen (0.5 mL loop, 5.0 mol% H_2_ diluted in Ar) were injected into the microreactor. The hydrogen consumption was monitored by a TCD detector (TCD3, Valco, Houston, TX, USA).

### 3.3. Catalytic Studies

Calcined hydrotalcites were studied as catalysts for ammonia decomposition to hydrogen and nitrogen. Prior to the catalytic run, the catalyst sample of 100 mg (catalyst’s grains fraction of 160–250 μm) was placed in a fixed-bed flow quartz microreactor and reduced in the flow of gas mixture containing 5.0 mol% H_2_ diluted in Ar (purity class 5.0) at 800 °C for 12 h. The microreactor was cooled down, also in a flow of H_2_/Ar gas mixture, to 250 °C. The catalytic tests were conducted in the range of 200–800 °C with the isothermal steps every 25 °C. The reaction mixture, supplied to the microreactor with a flow rate of 50 mL/min, is composed of 1.0 mol% ammonia diluted in helium (5.0). The progress of the reaction, ammonia conversion and formation of nitrogen and hydrogen, was continuously monitored by a quadrupole mass spectrometer (UMS TDS; PREVAC, Rogów, Poland) connected directly to the reactor outlet via a heated line. Catalytic tests were conducted under the space velocity of 300 mL/(h·g_cat_).

Moreover, a long-term isothermal stability test was conducted for the MZ03 catalyst at 450 °C for 23 h. The composition and flow rate of the reaction mixture were the same as in standard catalytic runs.

## 4. Conclusions

Hydrotalcite-derived Ni-Mg-Al metal oxide systems, with various Mg/Al ratios, were used as precursors of the catalysts for ammonia decomposition to hydrogen and nitrogen. It was demonstrated that calcination of ammonia in air as well as nitrogen results in the formation of periclase with incorporated nickel and aluminum cations, spinel phases and possibly also highly dispersed NiO. The reduction of the catalysts, which is the final step of their activation, resulted in the segregation of nickel species from different phases and the formation of nanometric particles of metallic nickel. It was shown that the size of such nickel particles was nearly constant in the subsequent reduction–oxidation cycles. Moreover, the results of H_2_-TPR clearly show that such nickel aggregates are reduced at significantly lower temperatures than nickel cations incorporated into particles and spinel phase, indicating relatively weak interaction of nickel particles with support. Thus, the real form of the catalysts operating under conditions of the catalytic reaction is nanometric nickel particles deposited on Mg-Al oxide support. The hydrotalcite-derived catalysts presented promising results of the catalytic studies with respect to their activity and stability, which increased with an increase in nickel content. The comparison of the Ni-Mg-Al hydrotalcite-derived catalyst and supported Ni/MgO and Ni/Al_2_O_3_ catalysts with similar nickel content shows the operation of the first catalyst at significantly lower temperatures. It was explained by significantly lower metallic nickel particles (about 10 nm) compared to other mentioned catalysts (above 20 nm) and, therefore, more space for nitrogen recombination and desorption, which is postulated to occur on the nickel particle–support borders.

## Figures and Tables

**Table 1:** Intended chemical composition of the catalyst precursors.

Sample	Intended Composition/mol%			
	Ni	Mg	Al	CO_3_^2−^
MZ01	5	55	40	20
MZ02	10	50	40	20
MZ03	20	40	40	20

**Table 2:** Chemical composition of the calcined (600 °C) hydrotalcite-like materials and specific BET surface area determined for the samples calcined at 600 °C and 800 °C as well as related catalysts after a single catalytic test.

Sample	Determined Composition/mol%			BET Surface Area/m^2^/g		
	Ni	Mg	Al	600 °C	800 °C	After Test
MZ01	5.2	54.0	40.8	313	204	202
MZ02	10.2	48.6	41.2	311	205	201
MZ03	20.2	38.5	41.3	334	200	171

**Table 3:** Nickel surface area (determined by the H_2_-chemisorption method) and nickel surface area related to nickel content.

Sample	Ni Surface Area /m^2^/g	Ni surface Area/Ni Content /m^2^/(g∙mol %)
MZ01	2.2	0.42
MZ02	4.2	0.41
MZ03	8.4	0.42

## Data Availability

Experimental data will be available for the request.

## Supplementary Materials

The following supporting information can be downloaded at https://www.mdpi.com/article/10.3390/molecules30051052/s1, Figure S1: Results of catalytic tests for hydrotalcite-like materials calcined at 600 °C. Catalyst’s composition (mol %): Ni-Mg-Al (10-49-41), Co-Mg-Al (10-50-40), Fe-Mg-Al (10-50-40).
