# Supplementary material for: Ni-Mg-Al Hydrotalcite-Derived Catalysts for Ammonia Decomposition—From Precursor to Effective Catalyst
Source: Molecules. 2025 Feb 25;30(5):1052. doi: 10.3390/molecules30051052 (PMC11901857; doi:10.3390/molecules30051052)
Supplement: Supplementary file 1 [file molecules-30-01052-s001.zip › molecules-3468285-supplementary.pdf]

Supplementary Materials

Catalytic studies

Calcined hydrotalcites were studied as catalysts for the ammonia decomposition to hydrogen and nitrogen. Prior to the catalytic run, the catalyst sample of 100 mg (catalyst's grains fraction of 100–160 µm) was placed in a fixed-bed flow quartz microreactor and reduced in the flow of gas mixture containing 5.0 mol% H_2 diluted in Ar (purity class 5.0) at 800 °C for 12 h. The microreactor was cooled down, also in a flow of H_2/Ar gas mixture, to 250 °C. The catalytic tests were conducted in the range of 200–800 °C with the isothermal steps every 25 °C. The reaction mixture, supplied to the microreactor with a flow rate of 50 mL/min, composed of 1.0 mol% ammonia diluted in helium (5.0). The progress of the reaction, ammonia conversion and formation of nitrogen and hydrogen, was continuously monitored by a quadrupole mass spectrometer (UMS TDS; PREVAC, Poland) connected directly to the reactor outlet via heated line. Catalytic tests were conducted under the space velocity of 300 mL/(h·g_{cat}).

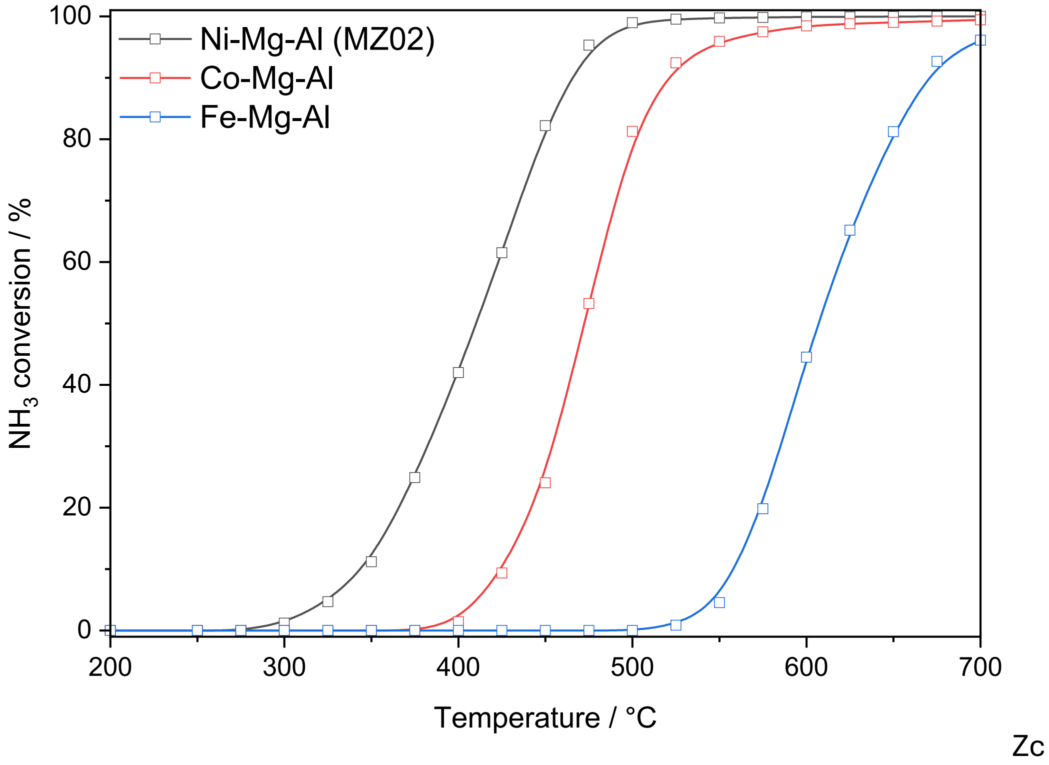

Figure S1. Results of catalytic tests for hydrotalcite-like materials calcined at 600 °C. Catalyst's composition (mol %): Ni-Mg-Al (10-49-41), Co-Mg-Al (10-50-40), Fe-Mg-Al (10-50-40).
